# The Impact of Periodontal Disease on Preterm Birth and Preeclampsia

**DOI:** 10.3390/jpm14040345

**Published:** 2024-03-26

**Authors:** Panagiotis Tsikouras, Efthymios Oikonomou, Konstantinos Nikolettos, Sotiris Andreou, Dimitrios Kyriakou, Christos Damaskos, Nikolaos Garmpis, Vassiliki Monastiridou, Theopi Nalmpanti, Anastasia Bothou, George Iatrakis, Nikolaos Nikolettos

**Affiliations:** 1 Department of Obstetrics and Gynecology, Democritus University of Thrace, 68100 Alexandroupolis, Greece; eftoikonomou@outlook.com (E.O.); k.nikolettos@yahoo.gr (K.N.); soterisand@hotmail.com (S.A.); dimitriskyrkdk@gmail.com (D.K.); basomonasteridou@gmail.com (V.M.); theonalmpanti@hotmail.com (T.N.); nnikolet@med.duth.gr (N.N.); 2 Department of Laboratory of Experimental Surgery and Surgical Research, Medical School, National and Kapodistrian University of Athens, 11527 Athens, Greece; x_damaskos@yahoo.gr; 3 Renal Transplantation Unit, Laiko General Hospital, 11527 Athens, Greece; 4 Department of Surgery, Sotiria Hospital, 11527 Athens, Greece; nikosg22@hotmail.com; 5 Neonatal Department, University Hospital Alexandra, 11528 Athens, Greece; natashabothou@windowslive.com; 6 Department of Obstetrics and Gynecology, National and Kapodistrian University of Athens, 11528 Athens, Greece; giatrakis@uniwa.gr; 7 Department of Obstetrics and Gynecology, Rea Maternity Hospital, 17564 Athens, Greece

**Keywords:** periodontal disease, pregnancy outcome, adverse effect

## Abstract

This review delves into the possible connection between periodontitis and negative pregnancy outcomes, such as preeclampsia and preterm birth. It highlights the potential influence of an unidentified microbial factor on preeclampsia and the effects of inflammatory responses on the rate of preterm births. Furthermore, it underscores the prevalent occurrence of oral ailments within the populace and their significant repercussions on quality of life. Hormonal fluctuations during pregnancy may exacerbate oral conditions such as pregnancy gingivitis and periodontitis, necessitating bespoke therapeutic approaches that take into account potential fetal ramifications. Periodontal disease, characterized by microbial attack and inflammatory response, results in tissue destruction and tooth loss. The oral cavity’s susceptibility to bacterial colonization, which is primarily due to its role as a site for food intake, is highlighted. Furthermore, research indicates a correlation between inflammatory responses and factors such as prostaglandin E2 and IL-1β, and preterm birth. Therapeutic interventions are a focus of international research, with efforts being aimed at optimizing outcomes through larger studies involving pregnant women.

## 1. Introduction

The association between periodontal diseases and adverse pregnancy outcomes, including premature birth, low birth-weight infants, and preeclampsia, has garnered significant attention in recent years [1,2]. Despite the established correlation, oral health remains an overlooked aspect of maternal care, with women often neglecting dental visits and preventive measures. A survey conducted among 520 pregnant women in Greece revealed that, while 74% perceived their dental health as satisfactory, a notable proportion, encompassing 237 participants, deferred dental care, citing reasons ranging from intentional avoidance to emergent circumstances. This reluctance may stem from a prevalent perception of periodontal disease, a well-established oral health issue which leads to severe complications in tooth support and preservation, particularly in its advanced stages.

Periodontitis, characterized by microbially induced chronic inflammation, constitutes a significant public health challenge, with profound implications for oral health and overall well-being. Surprisingly, a substantial portion of the individuals affected by periodontitis, estimated at 57%, downplay the significance of their symptoms, potentially delaying appropriate intervention [3]. The multifactorial etiology of periodontal disease involves a complex interplay between microbial toxins and host immune responses, exacerbated by various endogenous and exogenous factors, such as smoking, diabetes, AIDS, and stress, which accelerate disease progression [4,5,6].

Emerging research underscores the broader systemic implications of periodontal disease, positioning it within the framework of “periodontal pathology”. This paradigm shift highlights the interconnectedness between oral health and systemic conditions, including atherosclerosis, cardiovascular diseases, and pregnancy-related complications. Notably, evidence suggests a causal relationship between periodontal diseases and adverse pregnancy outcomes, with implications for maternal and neonatal health [6,7,8,9,10]. However, related findings were not identical among these studies due to the fact that heterogeneity was high among included populations. Against this backdrop, this paper aims to conduct a comprehensive literature review focusing on the intricate relationship between periodontal diseases and pregnancy complications, particularly the occurrence of premature birth and preeclampsia. By delving into the potential mechanisms underlying these connections, this review seeks to elucidate the pathophysiological pathways linking periodontal disease to adverse pregnancy outcomes. and estimate the pregnancy outcomes after treatment. Furthermore, by highlighting primary treatment strategies employed in addressing periodontal diseases during pregnancy, this paper endeavors to inform clinical practice and facilitate the development of evidence-based interventions aimed at optimizing maternal and neonatal health outcomes. However, it should be emphasized that there is no strong evidence that treatment of periodontal disease improves pregnancy outcomes [11].

## 2. Gingival Fissure

The tooth, unique in its capacity to penetrate the epithelial barrier yet incapable of proliferation or apoptosis, serves as a stable surface for microbial attachment and growth. Emerging from the gumline, it creates the gingival slit, with the gingival fissure separating it from the epithelium, typically with a depth of 0.5–3 mm. Termed the bottom and the gingival margin, respectively, the deepest and highest points of the fissure delineate its extent [12,13]. Subgingival plaque resides within the gingival crevice, contrasting with supragingival plaque outside it. The paragingival epithelium at the fissure’s base adheres to a healthy periodontium, while a depth exceeding 4 mm indicates pathological gingival sulcus, indicative of periodontitis and the subsequent formation of a periodontal pocket, fostering anaerobic microbial proliferation.

Periodontal disease arises from disrupted homeostasis, impacting connective tissue collagen production and osteoblast/osteoclast activity, leading to bone destruction. Recent perspectives attribute this disruption to an oral microbiome shift from symbiosis to dysbiosis, evoking an exaggerated immune response. Generalized periodontal disease may extend to 50 cm^2^, persisting untreated and causing epithelial lysis, collagen degradation, and vascular dilation, facilitating microbial and toxin infiltration into the bloodstream. Circulating microbes and toxins trigger inflammatory cytokine release, inducing remote pathological conditions. While the exact systemic disease risk remains uncertain, periodontopathogenic microbes or their genetic material detected in cardiovascular tissues and amniotic fluid suggest a direct systemic impact [12,13,14,15].

Various ailments are linked to periodontal disease, including diabetes, metabolic syndrome, cardiovascular disease, cancer, renal disease, respiratory disease, Parkinson’s disease, dementia, obesity, rheumatoid arthritis, and pregnancy complications like premature birth, low birth weight, and preeclampsia [12,13,14,15].

## 3. “Gumitis” of Pregnancy

The predominant change in the oral cavity during pregnancy is the onset of pregnancy gingivitis, affecting 36`–100% of pregnant women [16,17,18,19]. Pregnancy induces increased vascular permeability, alterations in immune system responses, and shifts in dental microbial plaque composition. Clinically, this manifests as heightened gum inflammation, increased bleeding upon probing (with consistent microbial plaque levels), and expanded measurements of the gingival fissure upon probing, without concurrent adhesion loss or bone destruction. Meta-analyses from 2013 note a surge in gingivitis prevalence during the second and third trimesters, followed by postpartum decline, with peak occurrences reported in either the second or third trimester [20,21].

Pregnancy also impacts the intestinal microbiome, and features estrogen and progesterone receptors, predominantly estrogen receptor-β, likely influencing cellular function [22]. Elevated estrogen and progesterone concentrations affect neutrophil functionality, pivotal in microbiome–host balance. Periodontal disease, or periodontitis, is characterized by varying severity, ranging from mild to severe involvement, including deep pockets and periodontal abscesses, as well as tooth instability, mobility, and loss. These conditions stem from gum inflammation prompted by Gram-negative anaerobic microbes like Porphyromonas gingivalis, Bacteroides forsythus, Treponema denticola, Actinobacillus actinomycetemcomitans, and Campylobacter rectus, among others. The body’s immune response is activated by microbial components such as lipopolysaccharide (LPS), binding to LPS-binding protein in the gums and forming a complex that subsequently activates macrophages. Inflammatory mediators like prostaglandin E2, IL-1β, and TNFα are released upon monocyte-derived macrophage activation, contributing to periodontal tissue degradation [21,22,23]. Elevated progesterone and estrogen levels exert effects on neutrophils, periodontal tissues, and the microbiota.

## 4. Preeclampsia Etiology

The precise cause of preeclampsia remains uncertain [24]. Nonetheless, it is theorized that inadequate placental implantation during early pregnancy may be linked to preeclampsia. The symptoms of preeclampsia, such as arterial hypertension, oxidative stress, and proteinuria, are believed to stem from a generalized dysfunction of the pregnant endothelium. Redman et al. proposed that inflammation triggers endothelial dysfunction, but it is unclear whether inflammation is a consequence or a precursor of preeclampsia [25,26]. Preeclampsia has been associated with deficient remodeling of spiral arteries, a process that begins in the first trimester and concludes by 18–20 weeks of gestation [27].

Redman suggested that inadequate spiral artery remodeling in early pregnancy results in insufficient placental growth, compromised perfusion, and oxidative stress, culminating in preeclampsia. However, this model fails to explain cases where poor placentation and fetal growth restriction occur without preeclampsia. Roberts modified Redman’s model, proposing that the imbalance between increased perfusion demands during pregnancy and maternal predisposition precipitates preeclampsia [28]. This implies that preeclampsia may arise from heightened uteroplacental demands without significant maternal predisposition, or from substantial maternal predisposition despite modest demands. Factors increasing perfusion demands include elevated placental volume, increased placental mass (e.g., twin pregnancy), and uteroplacental insufficiency. Maternal predisposing factors encompass genetic predisposition, obesity, advanced maternal age, hypertension, and endothelial damage.

Biochemically and at the cellular level, preeclampsia is associated with antiangiogenic responses (elevated sFLT1 and soluble endoglin, and decreased PLGF), abnormal trophoblast invasion, failed spiral artery remodeling, impaired oxygenation (increased HIF1α and oxidative stress), and compromised maternal immune responses (elevated inflammatory cytokines IL-6 and TNFα, and decreased anti-inflammatory cytokine IL-10). Kell and Kenny suggest that these factors may be triggered by microbial infection [28,29].

## 5. Periodontitis and Preeclampsia

The potential link between preeclampsia and an unidentified microbial factor, combined with evidence that periodontitis can alter microbiota and heighten risks of cardiovascular disease, atheromatous plaques, and endothelial damage, spurred scientific investigation into a potential correlation between periodontitis and preeclampsia. The literature suggests a clear association between periodontitis and increased preeclampsia risk [25,30,31,32,33]. Sgolastra et al., in a 2013 meta-analysis, found that periodontal disease elevates preeclampsia risk by 117% (OR 2.17) [31,34]. Moreover, Matevosyan et al., in a 2011 meta-analysis of 125 randomized trials, patient-control studies, and prospective studies (total sample: 992 births), revealed that women with preeclampsia exhibited poorer periodontal health across both intervention and control groups (OR 1.94–2.9). Elevated attachment loss (OR 2.76) was associated with increased preeclampsia incidence (RR 2.75), even after stratification for factors like gestational age, smoking, medical visits, and anemia [32].

Although the association of periodontitis with pregnancy complications is weaker compared to other factors, such as paternity, gestational age, and medical history [32], elevated levels of CRP and fetal tyrosine kinase increase preeclampsia risk [32]. Periodontitis raises systemic CRP levels [15]. While most studies on periodontal disease and pregnancy complications are epidemiological (89.6%), the intervention studies are scarce (10.4%), employing methods like debridement, root planning, mechanical biofilm removal, antibiotics use, and experimental periodontitis induction in animals. Vergnes’ 2008 systematic review involving 3420 births found that periodontal disease increased preeclampsia risk by 76% (OR 1.76, 95% CI: 1.43–2.18) [33]. In Conde-Agudelo et al.’s meta-analysis focused on epidemiological studies, it is documented that preeclampsia risk escalates among women with genitourinary system infections (OR 1.57; 95% CI, 1.45–1.70) or periodontal disease (OR 1.76; 95% CI, 1.43–2.18). However, no association was found between preeclampsia and various infections [33].

While epidemiological studies imply a link between periodontal disease during pregnancy and preeclampsia, intervention studies fail to show decreased preeclampsia risk after periodontal treatment in pregnant women. This discrepancy was noted in Kunnen et al.’s 2010 systematic review, which highlighted that though eight out of twelve epidemiological studies showed an association between periodontitis and preeclampsia, none of the three randomized intervention studies demonstrated reduced preeclampsia incidence following periodontal treatment during pregnancy [35]. For instance, Newnham et al.’s 2009 randomized study of 1082 pregnant women found no statistically significant reduction in preeclampsia risk with periodontal treatment at the twentieth week of pregnancy (4.1% for control, 3.4% for intervention; OR 0.82, 95% CI 0.44–1.56, *p* = 0.12). Periodontitis was defined as the presence of pockets (4 mm deep or more) in 12 or more fully erupted tooth spots, with treatments including microbial biofilm disruption, debridement, radical curettage, and correction of blockages [36,37]. It was postulated that there was a stronger relation of periodontitis with late-onset severe preeclampsia [38].

## 6. Mechanism of Association of Periodontitis and Preeclampsia

Two theories have emerged regarding the connection between periodontitis and preeclampsia, and it is plausible that these mechanisms operate concurrently, complementing each other. The first theory proposes that bacteremia associated with periodontitis leads to colonization of the embryo–placental unit by periodontopathogenic microbes, triggering an inflammatory response that culminates in preeclampsia induction. Conversely, the second theory suggests that cytokines released by the periodontium enter circulation, subsequently reaching the fetoplacental unit, where they elevate local inflammatory cytokine levels, tipping the balance towards preeclampsia induction. Alternatively, these cytokines may reach the liver, prompting a systemic inflammatory response, with resultant products reaching the fetoplacental unit and exacerbating local inflammation, leading to endothelial damage and preeclampsia [39].

Moreover, both periodontitis and preeclampsia occur against a genetic backdrop of immune hyper-responsiveness, suggesting a shared genetic predisposition for both conditions. Additionally, periodontal disease may serve as an indicator of detrimental habits that impact the pregnancy outcome, though a causal relationship remains undetermined. In other words, pregnant women with periodontal disease due to poor oral hygiene may be more prone to habits detrimental to pregnancy, increasing the risk of preeclampsia. The distribution of periodontal-secreted cytokines in plasma exacerbates inflammation and contributes to preeclampsia [39].

Another proposed mechanism linking periodontitis and preeclampsia involves the release of inflammatory mediators and cytokines by the periodontium, leading to their dispersion in plasma. These mediators, traveling through the bloodstream, initially affect the fetoplacental unit, amplifying local inflammatory factors, and subsequently stimulate a systemic inflammatory response in the liver. Products of this response re-enter circulation, further increasing local inflammation in the fetoplacental unit and contributing to endothelial damage and preeclampsia [26,39,40].

Studies have consistently reported elevated levels of various cytokines in preeclampsia, including PGE2, TNF-α, IL-1, IL-6, and CRP. However, inconsistencies exist regarding the specific cytokines implicated at different stages of pregnancy [41]. For instance, Kumar et al.’s 2014 prospective study found significantly higher plasma TNF-α and IL-4 levels in pregnant women with periodontitis compared to those with healthy gums, with lower TNF-α levels observed in those who subsequently developed preeclampsia [42].

While associations between periodontitis and preeclampsia have been established, the impacts on systemic inflammation and plasma cytokine levels remain inconsistent across studies. Although elevated CRP levels in periodontitis have shown greater consistency and significance, data regarding the relationship between pregnancy complications and inflammatory mediator levels in gingival fluid and plasma are limited [39,43,44,45,46,47,48,49]. Despite this, elevated levels of pro-inflammatory cytokines in plasma and circulating microbes stimulate the liver to produce fibrinogen and CRP, further exacerbating systemic inflammation [39].

Elevated levels of C-reactive protein (CRP) have been linked to pregnancy complications like gestational diabetes and preeclampsia [39]. CRP, serving as a marker of systemic inflammation, has been found in the research to be associated with various factors, including smoking, obesity, elevated levels of triglycerides, and periodontal disease [15].

Salberg et al. demonstrated that patients with rapidly progressive generalized periodontitis exhibited significantly higher CRP levels compared to those with localized rapidly progressing periodontitis and periodontitis-free control subjects [50]. The CRP levels reported in the study were 3.72 mg/L, 2.57 mg/L, and 1.54 mg/L, respectively. This evidence underscores the plausible inference that the presence of larger inflamed surfaces due to periodontal disease leads to heightened systemic inflammation levels, bolstering the likelihood of a causal association [50].

These data led to the conclusion that periodontitis heightens the risk of preeclampsia by contributing to increased systemic inflammation levels, thereby elevating circulating CRP levels and ultimately fostering heightened inflammation in the fetoplacental unit, leading to preeclampsia occurrence. While the association between periodontitis and increased plasma CRP levels is more robust than that of the aforementioned cytokines, further evidence is required in the literature to validate this mechanism. According to Kushner et al., slight increases in CRP levels (3–10 mg/L) are observed in approximately one-third of the population, potentially indicating cellular stress unrelated to inflammatory conditions, influenced by diverse factors such as dietary habits, altitude of residence, upper respiratory infections, and various medical conditions [51]. Some of these factors, including age, socioeconomic status, hypertension, diabetes mellitus, and obesity, affect both the occurrence of preeclampsia and CRP levels [51].

## 7. Association of Periodontal Disease with Preterm Birth

The potential link between periodontal disease and preterm birth has been extensively studied by multiple research groups, with numerous studies demonstrating correlations between periodontal disease, prematurity, and low birth weight. However, clinical investigations examining the impact of periodontal therapy on pregnancy outcomes have yielded mixed results. These discrepancies can be attributed to at least two factors: antimicrobial drugs’ inability to fully reverse the adverse effects of periodontal pathogens colonizing the embryo–placental region, which occurs late in the first trimester, and considerable variation among studies in terms of population demographics, definitions of periodontitis, and risk factors covered, leading to conflicting findings [52].

In a Taiwanese study, periodontal disease presence in expectant mothers was associated with an increased likelihood of premature birth, with disease severity positively correlating with preterm delivery risk, particularly in women aged 31 to 35 years and those with higher economic status [53]. Similarly, in a Brazilian study, approximately 10% of women with periodontal disease experienced preterm labor, emphasizing its significance as an infection linked to preterm birth, alongside urinary tract infections. Marital status also played a role in preterm birth occurrence [54]. A Brazilian case-control study has found that the combination of periodontitis and hypertension could quadruple the risks of premature birth and low birth weight, although microbiological analyses revealed limited associations between preterm delivery, low birth weight, and most periodontal pathogens examined [55].

Similarly, a research effort conducted in Rwanda revealed that periodontitis significantly elevated the chances of premature birth, by six times, compared to women without periodontitis, irrespective of maternal age [56]. Another study by De Oliveira et al., conducted in Brazil and involving 2474 participants, reported a preterm birth rate of 10.2%, with a 21.7% prevalence of gingivitis and 14.9% of periodontitis among pregnant women [57]. Those with periodontitis demonstrated nearly twice the risk of preterm birth compared to their healthy counterparts, with an escalated risk of early preterm delivery associated with the presence of periodontal pockets of 5 mm or more and bleeding upon examination. However, in a Spanish study, Gallagher-Cobos et al. did not find statistically significant correlations between maternal periodontal disease and preterm birth or low birth weight. Similarly, no significant associations were observed with different dental techniques. Smoking during pregnancy was associated with increased rates of low birth weight but not with preterm birth incidence [58]. However, it should be noted that preterm delivery was found to be associated with use of heated tobacco products during pregnancy [59].

## 8. Periodontal Diseases and Preterm Birth—Mechanisms

Today, it is recognized that a substantial proportion of premature and low birth weight (PLBW) cases, ranging from 30% to 50%, can be attributed to infections, with periodontitis and periodontal diseases representing genuine oral infections. Infection has emerged as a primary contributor to low birth weight and preterm births [60]. The oral cavity serves as a continual source of infectious agents, and alterations in its health often indicate underlying diseases. Serving as a bacterial reservoir, periodontal infection holds the potential to exacerbate systemic disorders. Research indicates that the bacteria accountable for gum inflammation have the capability to enter the bloodstream and impact the developing fetus, possibly resulting in premature delivery and reduced birth weight in offspring. Endotoxins generated by Gram-negative bacterial infections such as periodontal disease represent the inception of a plausible pathway in this regard.

Proinflammatory mediators can breach the placental barrier, inducing fetotoxicity, resulting in preterm delivery and low birth-weight neonates. These endotoxins also stimulate the release of cytokines and prostaglandins (IL-1, IL-6, and TNF-), which, in appropriate concentrations, trigger labor [60,61].

High levels of these cytokines in pregnant women can lead to uterine membrane rupture, precipitating premature birth and developmental abnormalities. There is an urgent need to explore the potential correlation between adverse pregnancy outcomes and periodontal diseases, particularly as periodontal therapy remains relatively underdeveloped in Asia. This correlation, if established, could have implications for preventive oral health programs integrated into prenatal care for expectant mothers.

Multiple animal and clinical studies have demonstrated a strong association between periodontal infection and adverse pregnancy outcomes. A theoretical framework envisions persistent periodontal infection mediating this systemic effect through several processes. However, a clear causal relationship has yet to be established, and alternative explanations for the association can be proposed. The European Federation of Periodontology/American Academy of Periodontology joint laboratory consensus report on periodontitis and systemic diseases delineates two major pathways for the biological mechanisms of adverse pregnancy outcomes linked to oral pathogens: direct mechanisms, where oral microorganisms invade the placenta and amniotic cavity via blood-borne dissemination or an ascending route through the genitourinary tract, and indirect mechanisms, fueled by inflammatory mediators produced in response to pathogenic microorganism invasion in periodontal tissues. These mediators may directly impact the fetoplacental unit or circulate to the liver, amplifying the systemic inflammatory response, which may subsequently affect the fetoplacental unit [52]. It has been proposed that periodontal disease causes a temporary bacteremia that aids in transporting viable microbes across the placenta. Some premature infants have exhibited humoral immune responses against microorganisms associated with periodontal disease, supporting this hypothesis. Oral bacteria such as *F. nucleatum* and *P. gingivalis* have been identified in preterm amniotic fluid and placenta samples. However, it is important to note that certain oral bacteria linked to preterm labor are commonly found in the vagina, including *F. nucleatum*, *Peptostreptococcus* spp., *Streptococcus* spp., *Dialister* spp., and *Veillonella* spp. Therefore, their presence in the intra-amniotic region does not necessarily indicate an oral transmission route. Nevertheless, strong clinical and genomic evidence suggests the possibility of some intrauterine infections originating orally [62].

Han et al. detected an uncultured strain of Bergeyella in the amniotic fluid of a woman diagnosed with chorioamnionitis through analysis of the 16S rRNA gene. Interestingly, the same strain was identified in the patient’s sublingual plaque, but was absent in her vaginal canal, suggesting an oral transmission route [63].

Pregnancy is accompanied by changes in periodontal health, with expectant mothers being more susceptible to inflammatory conditions and experiencing increased gum bleeding rates due to immune system changes. This suggests that for women with pre-existing periodontitis before conception, pregnancy could exacerbate the condition’s severity [64]. Early pregnancy is often characterized by pregnancy-induced nausea and vomiting, during which gastric acid production may compromise periodontal tissue barriers to various microorganisms, potentially contributing to placental infection and systemic inflammation, ultimately resulting in preterm labor [64]. Additionally, hormonal fluctuations during pregnancy may alter the oral biofilm composition, potentially aggravating gingival inflammation. Researchers suggest that the progression of pathological processes may be facilitated by hematogenous dissemination of bacteria and pro-inflammatory mediators from periodontal infection sites to the placenta, fetal membranes, and amniotic cavity.

However, opinions among researchers remain divided, with some studies supporting this theory, while others fail to demonstrate that treating periodontal disease during pregnancy improves neonatal outcomes [52].

The release of periodontal-secreted cytokines into the plasma due to inflammation can lead to premature birth or low birth weight. During pregnancy, the levels of inflammatory cytokines such as IL-1β, TNF-α, and PGE2 in amniotic fluid increase to critical levels, precipitating amniotic sac membrane rupture, uterine contraction, cervical dilation, and ultimately the onset of labor [60]. Elevated amniotic fluid levels of IL-1β, IL-6, TNF-α, PGE2, fibronectin, and α-fetoprotein have been associated with preterm birth [64,65,66,67]. MMPs, including MMP-2, MMP-8, MMP-9, and MMP-13, also play a significant role in the mechanism of preterm labor induction [68]. Infections and inflammations have been linked in the literature to pregnancy complications such as premature birth and can affect the systemic circulation levels of the aforementioned inflammatory factors. Increased plasma levels of IL-1, IL-6, IL-8, TNF-α, and CRP have been associated with premature or low birth-weight births [39,67,69,70,71,72]. Periodontal patients also exhibit increased pro-inflammatory cytokines and inflammatory mediators. These factors, stemming from periodontal tissue inflammation, can diffuse into gingival fluid or enter the circulation [39]. Increased plasma levels of IL-1β, IL-6, TNF-α, CRP, MMP-8, and MMP-9 have been observed in patients with periodontitis [39,67,69,70,71,72].

Plasma levels of TNF-α and PGE2 were found to be elevated in women with periodontitis compared to periodontally healthy women, while pregnant women with periodontitis showed elevated plasma levels of TNF-α, IL-4, and IL-6 [39,67,69,70,71,72]. Salivary and plasma levels of IL-1β, IL-6, and TNF-α increase with periodontal disease severity [47], and plasma MMP-8 and MMP-9 levels decrease after non-surgical periodontal treatment events, supporting the theory of periodontitis causing increased systemic inflammation. The correlation between TNF-α levels in gingival fluid and PGE2 levels in plasma further reinforces this notion [47].

The theory of the indirect effects of periodontitis on premature or low birth-weight births involves elevated levels of matrix metalloproteinase-8 (MMP-8) and matrix metalloproteinase-9 (MMP-9) [68]. However, periodontal therapy during pregnancy does not appear to significantly improve plasma inflammation markers, except for CRP levels, MMP-8, and MMP-9 levels. Moreover, it does not consistently improve inflammation markers derived from cord blood, and there is only inconsistent evidence that it reduces the risks of low birth weight or preterm birth [66,67,68]. The observation that periodontal treatment does not mitigate the risk of preterm birth or low birth weight may prompt a shift in research focus toward considering the likelihood that the association between periodontitis and adverse neonatal outcomes primarily operates through a direct pathway. This pathway involves the migration of periodontopathogenic microbes through the bloodstream to the placenta, microbes which, according to studies in experimental animals, are capable of locally increasing concentrations of TNF-α, IL-1β, MMP-2, and MMP-9. Meanwhile, the indirect pathway likely plays a secondary and complementary role [39].

## 9. Coping Strategies

There is currently no established evidence-based guideline for treating periodontal infections during pregnancy. The primary objective of periodontal treatment in the general population is to diminish periodontal tissue infection through meticulous and consistent oral hygiene education, medical management, and mechanical (surgical) intervention. Systemic antibiotics are included in the treatment regimen for severe, chronic, or aggressive forms of periodontitis [52].

Dental care recommendations for expectant mothers vary among different countries. Some countries incorporate oral hygiene guidance and treatment advice into postpartum care standards. In the United States, only 34% of pregnant women seek dental care during pregnancy. To mitigate potential complications during pregnancy, pregnant women, obstetricians, and dental professionals exercise caution when recommending specific dental treatments. Moreover, approximately half of obstetricians would not routinely suggest a dental check-up unless national guidelines explicitly endorse it. Even when pregnant patients are referred to dentists, only 10% of practitioners complete all necessary procedures, and 14% are hesitant to administer local anesthetics during pregnancy [73].

A meta-analysis conducted by the Cochrane Oral Health Group examined the efficacy of addressing periodontal disease in expectant mothers for the purpose of preventing or mitigating neonatal and maternal morbidity and mortality. The analysis encompassed 15 randomized controlled trials (RCTs) with the participation of 7161 subjects [74]. Researchers compared periodontal treatment with alternative interventions, and periodontal treatment during pregnancy versus no treatment. The meta-analysis revealed no notable distinction between receiving periodontal treatment and receiving no treatment concerning preterm birth at 37 weeks. Consequently, the authors concluded that the effect of periodontal treatment during pregnancy on preterm birth remains uncertain. They emphasized the necessity for further investigation to determine the most effective periodontal treatment for preventing adverse pregnancy outcomes [74].

Research has indicated that addressing periodontal disease before the 21st week of pregnancy may help prevent preterm births. Although there are differing opinions, most studies suggest that treating periodontal issues during pregnancy is safe and can improve overall periodontal health. Thus, it is recommended that periodontal disease be detected and treated early, to decrease the likelihood of negative pregnancy outcomes and preterm births linked to periodontal issues. The most effective strategy for preventing oral disorders and their impact on pregnancy is preventive dental care and treatment before conception. Periodontal disease identified during pregnancy should be carefully managed to control it and plan any necessary interventions, in order to minimize the risk of preterm birth.

In addition to mechanical treatments, systemic antibiotic therapy with amoxicillin or metronidazole may be prescribed for severe periodontitis. Current research suggests that antibiotic use, particularly metronidazole, does not adversely affect pregnancy outcomes. The critical phases of fetal organogenesis, which are highly susceptible to external influences, particularly drugs, occur during the first 12 weeks of gestation. Therefore, during the first trimester, only emergency dental procedures are recommended. Dental plaque removal by a dentist should supplement oral hygiene maintenance. Elective dental care is considered safest during the second trimester if required. While certain treatment options are available during pregnancy, it is generally advisable to postpone extensive restorations or major surgical procedures until after childbirth [52]. The primary concern regarding systemic drug or antibiotic use during pregnancy is the potential for teratogenic effects. Dental professionals adhere to Food and Drug Administration (FDA) categorizations when prescribing medications to pregnant patients, based on the risk of adverse fetal outcomes [52].

## 10. Discussion

Premature or underweight births, along with the onset of preeclampsia, represent three significant complications during pregnancy. Premature birth stands as the primary cause of neonatal morbidity and mortality, affecting approximately one in ten pregnant women, with a rising incidence observed in developed nations, despite scientific efforts [63,64,65,66]. Although most premature infants survive today, they may face potential long-term health consequences [52]. The birth of an underweight newborn follows premature birth as the second leading cause of perinatal mortality [68]. Studies suggest that this occurrence is linked to an increased risk of type 2 diabetes in adulthood [55]. Preeclampsia affects 5–7% of pregnant women and remains a leading cause of maternal mortality in certain regions. For mothers, preeclampsia increases the likelihood of future cardiovascular conditions such as chronic hypertension, heart attack, or stroke, while premature birth due to preeclampsia heightens the newborn’s vulnerability later in life to stroke, cardiovascular diseases, and metabolic syndrome [75,76,77,78]. Despite extensive research, the precise causes of preeclampsia remain incompletely understood [41]. Conducting studies to probe into the origins of these pivotal pregnancy complications is imperative for enhancing our comprehension of pathogenetic mechanisms and facilitating improved preventive measures.

Periodontitis, characterized by chronic inflammation of microbial origin, progresses slowly and leads to gradual deterioration of tooth support structures. In cases of generalized periodontal disease marked by deep pockets, the inflamed area can encompass up to 50 cm². Inflammation induced by periodontal disease triggers epithelial lysis, collagen fiber degradation, and vascular dilation, resulting not only in bleeding but also in the infiltration of microbes and microbial toxins into the bloodstream. Certain microbes exhibit tissue-penetrating capabilities. Circulating microbes and their toxins can prompt liver cells to produce inflammatory cytokines, thereby instigating their secondary production by other cells.

Epidemiological studies have indicated that periodontitis can elevate systemic inflammation levels. Furthermore, periodontopathogenic microbes or their genetic material have been detected in cardiovascular tissues and amniotic fluid, areas inaccessible except via the bloodstream [22,79].

Given periodontitis’s potential to heighten systemic inflammation levels and trigger recurrent microbiota episodes, and the presence of periodontopathogenic microbes in remote sites such as amniotic fluid—along with the resemblance of the placental microbiome to that of the oral cavity—it is logical to explore the epidemiological association between periodontitis and systemic diseases and subsequently ascertain causality. Numerous studies furnish compelling evidence implicating periodontal disease in preterm birth. Additionally, findings from various investigations suggest that periodontal infections may disseminate from periodontal tissues to the placental site and cause infection. Nevertheless, the precise pathways facilitating inflammation propagation from the oral cavity to the placenta and contributing to adverse pregnancy outcomes remain elusive, necessitating further research. Additionally, there are abundant data underscoring the imperative of instituting a preventive program to mitigate pregnancy complications. Preventive measures encompass pre-conception counseling, early medical assessments, and minimizing bacterial plaque through specialized hygiene sessions, education, and promotion of sound dental and nutritional hygiene practices.

Research gaps and potential study designs can be identified in the context of the link between periodontitis and pregnancy complications. While this review outlines several associations between periodontal disease and adverse pregnancy outcomes, such as preterm birth and preeclampsia, there remains uncertainty about the precise pathways through which periodontal inflammation affects pregnancy. To address this gap, longitudinal cohort studies could be designed to follow pregnant individuals with periodontal disease, tracking their oral health status throughout pregnancy and assessing pregnancy outcomes. These studies could employ advanced imaging techniques to investigate potential mechanisms of microbial dissemination from periodontal tissues to the placenta. Additionally, there is a need for randomized controlled trials to evaluate the effectiveness of preventive measures, such as specialized hygiene sessions and dental interventions, in reducing the incidence of pregnancy complications in individuals with periodontitis. Furthermore, qualitative research could explore the cultural factors influencing the acceptance and uptake of personalized medical care interventions among pregnant individuals with periodontal disease, thus informing culturally competent care practices. Interdisciplinary collaboration between obstetricians and dentists is essential for designing and implementing these studies, ensuring comprehensive care and improved health outcomes for pregnant individuals.

Personalized medical care for pregnant individuals with periodontal disease encompasses a comprehensive approach tailored to address their specific health needs. This involves individualized interventions such as more frequent dental visits and specialized periodontal treatments, particularly for those with a history of preterm birth or preeclampsia.

Moreover, personalized medical advice extends beyond dental interventions to encompass holistic strategies that promote maternal and fetal well-being. This may include personalized dietary recommendations of foods rich in essential nutrients crucial for both dental and fetal development. Additionally, considerations of mental well-being, exercise, and stress management play vital roles in optimizing pregnancy outcomes.

Furthermore, cultural sensitivity is paramount in delivering personalized medical care. Healthcare providers should take into account patients’ preferences, beliefs, and cultural practices to ensure that interventions resonate with the patients’ individual values and lifestyles. Providing information in multiple languages, accommodating religious practices, and offering alternative treatment options when appropriate are essential aspects of culturally competent care.

By integrating personalized medical-related content into prenatal care discussions, healthcare providers can empower expectant mothers with periodontal disease to make informed decisions about their oral health and pregnancy. This individualized approach fosters trust, enhances patient–provider communication, and ultimately leads to improved maternal and fetal health outcomes.

Strengthening interdisciplinary collaboration between obstetricians and dentists is crucial for optimizing the health of pregnant women. Updating dental care standards, patient education, and healthcare worker training could substantially alleviate the burden of periodontal disease and the associated risk of preterm births. Future investigations aimed at validating the link between periodontal disease and preterm birth should adopt a comprehensive methodology, incorporating an accurate definition of the condition, robust control of preterm birth confounders, and effective periodontal infection eradication interventions.

## 11. Conclusions

We highly recommend that couples who are actively seeking to conceive, regardless of whether they are pursuing natural conception methods or assisted reproductive techniques, greatly benefit from undergoing comprehensive prenatal screening aimed at assessing oral hygiene. This proactive approach not only allows for the identification and management of any existing dental issues that could potentially impact fertility or pregnancy outcomes but also promotes overall maternal and fetal health. Moreover, to further enhance maternal well-being during pregnancy, it is proposed that dental care be seamlessly integrated into the antenatal monitoring process, particularly during the pivotal first trimester. Obstetricians play a crucial role in this integration by making recommendations for early dental assessments and care during the initial visit to the dentist, thereby ensuring that any dental concerns are promptly addressed and managed in tandem with the ongoing prenatal care regimen. This collaborative approach between obstetricians and dental professionals not only optimizes the oral health statuses of expectant mothers but also contributes to the comprehensive management of maternal and fetal well-being throughout the course of pregnancy. Prospective studies in populations with similar characteristics could reduce a variety of biases, such as the selection bias present in studies with differing designs and/or studies conducted in different populations. Finally, dental care might be integrated into prenatal care protocols. However, an essential step in this direction is the adoption of practical strategies by a broad spectrum of individuals implicated in education and public health [80].

## Data Availability

Data of study participants are saved in the Department Obstetrics and Gynecology of Democritus University of Thrace, Greece.
